# SLC38A10 Transporter Plays a Role in Cell Survival Under Oxidative Stress and Glutamate Toxicity

**DOI:** 10.3389/fmolb.2021.671865

**Published:** 2021-05-05

**Authors:** Rekha Tripathi, Tanya Aggarwal, Robert Fredriksson

**Affiliations:** Molecular Neuropharmacology, Department of Pharmaceutical Biosciences, Uppsala University, Uppsala, Sweden

**Keywords:** SLC38 family, SLC38A10, primary cortex cells, glutamate toxicity, oxidative stress, p53, cell survival

## Abstract

Solute carrier (SLC) transporters regulate amino acids, glucose, ions, and metabolites that flow across cell membranes. In the brain, SLCs are the key regulators of neurotransmission, in particular, the glutamate/GABA-glutamine (GGG) cycle. Genetic mutations in SLCs are associated with various neurodevelopmental and neurodegenerative diseases. In this study, we have investigated the role of SLC38A10 under acute oxidative and glutamate stress in mouse primary cortical cells from SLC38A10 knockout (KO) mice. The ER/golgi localized transporter, SLC38A10, transports glutamate, glutamine, and alanine in brain cells, and the aim of this study was to determine the possible effects of removal of SLC38A10 in primary cortical cells under glutamate and oxidative challenges. Primary cortical neuronal cultures of wild-type (WT) cell and SLC38A10 KO mice were subjected to different concentrations of glutamate and hydrogen peroxide. There was no morphological change observed between KO and WT cortical neurons in culture. Interestingly, KO cells showed significantly lower cell viability and higher cell death compared to WT cells under both glutamate and hydrogen peroxide exposure. Further, we evaluated the possible role of p53 in neuronal cell apoptosis in KO cells. We found decreased intracellular p53 protein levels under glutamate and hydrogen peroxide treatment in KO cortical cells. In contrast, caspase 3/7 activity remains unaltered under all conditions. These results demonstrate an indirect relationship between the expression of SLC38A10 and p53 and a role in the cell defense mechanism against neurotoxicity.

## Graphical Abstract

SLC38A10 role in cells survival under stress.

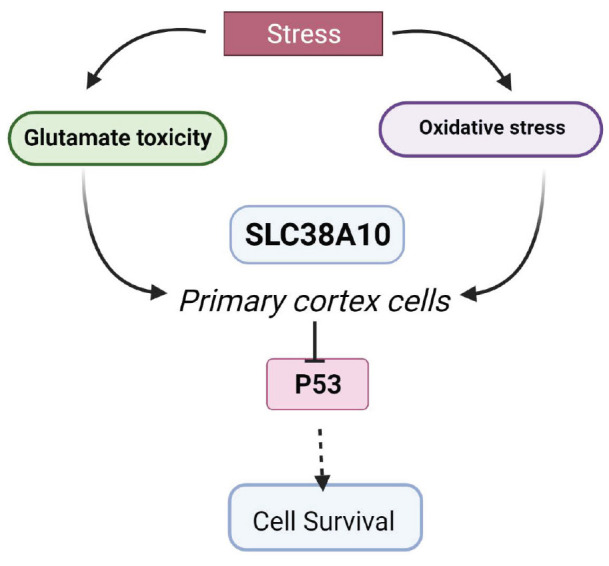

## Introduction

Neuronal cells are the key players in the nervous system, with highly specialized roles in exchanging and transmitting signals. Cells maintain homeostasis with estimated cell growth and death rate. In the body, cells undergo a series of external and internal changes, an act of survival and adaptation forced by various internal and external stressors. Stress can result in either the activation of defense mechanisms to protect cells or the activation of signaling cascades leading to cell death (Fulda et al., 2010).

Oxidative stress in neuronal cells results from the presence of excess oxygen radicals that can disrupt the homeostasis of the cells, which can lead to cell death. In normal conditions, cells require a balance of antioxidants and oxygen that maintains the equilibrium between antioxidant defense mechanisms and pro-oxidant species (Fulda et al., 2010). Under stress, an increase in reactive oxygen species (ROS), such as hydrogen peroxide (H_2_O_2_), hydroxyl radical, and superoxide anion, can affect the critical processes of cell health, such as cellular respiration, metabolism, immunity, and cell death (Fulda et al., 2010; Wang and Michaelis, 2010; Salim, 2017). Another stress discussed in this study is glutamate excitotoxicity, which occurs due to increased amounts of glutamate. It is one of the leading causes of neuronal death and mitochondrial dysfunction, ultimately leading to neurodegenerative diseases (Lewerenz and Maher, 2015; Fricker et al., 2018).

Stress stimuli induce cellular stress that can support cell survival or lead to cell death (Fulda et al., 2010). The cells can initiate different death signaling pathways, depending on the type of cellular stress response. The cell death program can vary from programmed cell death (apoptosis and autophagy) to necrosis or other forms of unknown responses (Fricker and Tolkovsky, 2011). Apoptosis is characterized biochemically by events of DNA damage, chromatin condensation, nuclear fragmentation, phagocytosis of apoptotic bodies, and activation of different caspases leading to cell death (Kerr et al., 1972). Autophagy is an internally regulated mechanism to remove or clean the unnecessary or degenerated cell material. It is mostly initiated when cells undergo metabolic stress like starvation of nutrients and growth factors, leading to ROS accumulation ultimately, affecting mTOR signaling (Lum et al., 2005; Fricker et al., 2018). Necrosis is an unnatural cell death due to various factors such as glutamate toxicity, ROS, and calcium generation, leading to swelling and gain of cell volume and rupturing of the plasma membrane (Ankarcrona et al., 1995; Schulze-Osthoff et al., 1998).

In cells, amino acid transporters play vital roles of sensors and transporters of nutrients, neurotransmitters, drugs, and ions (Sundberg et al., 2008). Most amino acid transporters are members of the solute carrier (SLC) family that consists of 460 members, of which many are poorly characterized (Perland and Fredriksson, 2017). SLCs are membrane-bound transporters that are divided into 65 families with different biochemical properties and substrates of transport (Hediger et al., 2013; Kandasamy et al., 2018). In the brain, 287 SLCs have been shown to be expressed in the brain (Hu et al., 2020) and around 72 SLC genes correlated with human brain disorders such as epilepsy, neurodegenerative diseases, and autism. SLCs in the brain play important roles in regulating the homeostasis of neurotransmitters, such as GABA, glutamate, serotonin, dopamine, and noradrenaline, and in the regulation of their concentration in synaptic regions. Therefore, it is important to investigate the functions of SLCs expressed in the brain linked to neurotransmission. In this study, we have focused on one member of the SLC38 family, SLC38A10, and some other members of this family are involved in regulating the glutamate and glutamine cycle in the brain, for example, SLC38A3 and SLC38A5 (Şehirli and Aykaç, 2020). The SLC38 amino acid transporter family is known as the sodium-coupled neutral amino acid transporter (SNAT) family, and it consists of 11 members, SNAT1-11 (Sundberg et al., 2008). Most of the members are expressed in the brain in various cell types (Hägglund et al., 2011, 2015). SLC38A10 is found in endoplasmic reticulum (ER)/golgi of neurons and astrocytes in the mouse brain and transports glutamine, glutamate, and aspartate (Hellsten et al., 2017; Tripathi et al., 2019). The functional relevance remains unexplored in terms of the effect on the health of brain cells. This study presents the effect of SLC38A10 gene knockout (KO) on primary mouse cortical cells under normal and stressed conditions. We found that SLC38A10 KO cells showed differences in cell viability, proliferation, and cytotoxicity compared to wild-type(WT) cells.

## Materials and Methods

### Animals

The study experiments using mice were approved by the Uppsala Animal Ethical Committee (3 5.8.18-09820/2018) and followed the Swedish welfare legislation. The animals were kept in an animal facility with a controlled environment, including humidity (45–65%), temperature (20–24°C), and a ventilation system with a 12-h light/dark cycle where they had access to food and water. C57BL/6 mice were used for both WTs and as a background for a KO line. KO mice were bought from IMPC^1^ with Slc38a10tm2a (EUCOMM)Wtsi/H allele. For making the embryonic primary cultures, male and female mice were put together. A check for a vaginal plug was carried out in the morning after. The day when a vaginal plug was detected was defined as gestation day e0.5.

### Primary Mouse Cortical Cells and Treatments

Primary cortical cultures were performed as described previously (Hellsten et al., 2018), e15 embryos were removed and kept in cold Hank’s Balanced Salt Solution (HBSS) (Gibco, United States). The mouse cortical tissue was dissected in 1X PBS with 10 mM glucose from WT and KO mice. The tissue dissociated using both chemical and mechanical procedures using an enzymatic mixture of DNAse (Invitrogen, United States) (1 μl) and papain (Sigma, United States) (10 μl) in 1 ml diluted in PBS-10mM glucose for 20–30 minutes at 37°C, 5% CO_2_. The tissue was replaced and mechanically dissociated in a plating media containing DMEM/F12 (Invitrogen, Thermo Fisher Scientific, United States), 10% FBS (Gibco, United States), 2 mM GlutaMax (Invitrogen, United States), 1 mM Na-pyruvate (Invitrogen, United States), and 1% Penicillin/streptomycin (Invitrogen, United States). The cell suspension was filtered through a 40-μm cell strainer and counted with a hemocytometer. The plates/coverslips (12 mm, Menzel Glaser) to be used were coated with poly-L-lysine solution (P4707, Sigma-Aldrich, United States). Cells were plated at densities of 10,000 cells/well for 96-well-plates, 50,000 cells/well for 24-well-plates, and 100,000 cells/well for 6-well-plates. Cells were incubated for 3 h at 37°C in 5% CO_2_. Once the cells were attached, the plating media (Gibco^®^, Life technologies, United States) was replaced with the media containing Neurobasal A media (Invitrogen, United States), 2% B27, 1% penicillin/streptomycin, 2 mM GlutaMax, and 1 mM Na-pyruvate. Two days after plating, half of the media was replaced with fresh media, followed by 7 days of culture.

For treatment, hydrogen peroxide (Sigma-Aldrich, United States, 386790-M, CAS Number 7722-84-1) was used to induce oxidative stress, and glutamate (Sigma-Aldrich, United States, G1251) was used to induce glutamate excitotoxicity. On day 7, the media was replaced with fresh media, and cells were treated with hydrogen peroxide (100 and 200 μM) or glutamate (100 and 500 μM) or without any treatment as controlled and incubated for 2 h at 37°C. After treatment, the cells were subjected to the experiments described below. All experiments were performed in triplicates.

### Measurement of Cell Viability

Cell viability was measured with CellTiter 96 Aqueous One Solution Cell Proliferation Assay (Promega G3582, United States), which is composed of tetrazolium compound MTS [3-(4,5-dimethylthiazol-2-yl)-5-(3-carboxymethoxyphenyl)-2-(4-sulfophenyl)-2H-tetrazolium]. MTS is bio-reduced by cells to a colored formazan product that is soluble in the culture media. The quantity of formazan measured is directly proportional to the number of living cells in culture. Cells were cultured in 96-well-plates as described above, and after hydrogen peroxide and glutamate treatment, 20 μl of CellTiter 96^®^ AQueous One Solution was added into each well containing 100 μl of the culture medium, and absorbance was measured at 490 nm using a plate reader. Results were presented as a measure of the relative fluorescence unit.

### Measurement of Cytotoxicity

Cytotoxicity was determined using CytoTox-Fluor Cytotoxicity (LDH) assay (Promega G9260, United States) according to the instructions of the manufacturer. The assay used the fluorogenic peptide substrate (bis-AAF-R110) to measure protease activity associated with cytotoxicity, which is proportional to amount of dead cells. Cells were grown in opaque-walled 96-well-plates for 7 days, followed by treatments. CytoTox-Fluor Cytotoxicity assay reagent (100 μl) was added to the cells with 100 μl of the media in each well. The plate was incubated for 30 min at 37°C, followed by fluorescence measurement using a fluorometer at 485 nmEx/520 nmEm, and the results were presented as a measure of relative fluorescence unit.

### Assay for Caspase 3 and Caspase 7

Caspase 3 and 7 activities were measured using a luminescent Caspase-Glo 3/7 Assay System (Promega G8090, United States). It is based on cell lysis, followed by caspase cleavage of the luminogenic caspase-3/7 substrate and generation of the luminescence signal produced by luciferase. Luminescence produced is proportional to the amount of caspase activity. Cells were cultured in white-walled 96-well-plates followed by the treatments as described previously. Each well containing 100 μl of the media was added with 100 μl of the Caspase-Glo 3/7 reagent. Cells and plates were incubated at room temperature for 1 h. Then, luminescence was recorded with a plate reader.

### Measurement of ATP Levels

ATP levels were measured using the Cell Titer-Glo Luminescent Cell Viability Assay (Promega G7570, United States) according to protocol of the manufacturer. The number of metabolically active cells in culture generates a luminescent signal directly proportional to the amount of ATP. Cortical cells were cultured in opaque-walled 96-well-plates. After treatment, 100 μl of the Cell Titer-Glo reagent was added to wells containing 100 μl of the media. The plate was incubated at room temperature for 10 min. Luminescence was recorded with the plate reader, and data were presented graphically compared to controls.

### JC1 Assay

Cells were cultured and treated as described above but in 24-well-plates. On day 7, the cell culture medium (with or without treatment) was removed and washed once with a fresh medium. Then, the medium was replaced with a medium containing JC1-dye 2 μM (Thermofisher, Cat No. 65-0851-38), and the cells were incubated for 20 min at 37°C. The medium was removed and replaced with PBS, and cells were imaged using ImageXpress Micro XLS microscope (Molecular Devices, United States) with FITC and TxRED filter. Cell profiler was used to analyze JC1 monomer (green) and JC1 aggregated (red) mean fluorescence intensity calculated per image. The ratio for red and green intensity was calculated and represented as mean with SD.

### Western Blot

Protein samples were extracted from mouse cortical cells after 7 days of culture and treatment. Samples were made by cell lysis in RIPA buffer, and protein concentration was measured using Bradford reagent (Sigma-Merck #B6916, United States) at 595 nm. SDS-PAGE was performed using 20 μg of protein sample diluted at 1:1 ratio in Laemmlis buffer (Bio-Rad Lab) with β-mercaptoethanol (Sigma, United States) and heat-treated at 95°C for 5 min. Samples were loaded on 8–12% SDS PAGE gel (Biorad, United States), and after electrophoresis, they were transferred to Trans-Blot Turbo Mini 0.2 μm Nitrocellulose (Biorad #1704158, United States) membrane. As a molecular weight marker, the prestained protein ladder of 10–180 kDa (Thermo Fisher Scientific, United States) was used. The membrane was blocked in 5% milk TTBS blocking solution for 1 h followed by overnight incubation with primary antibodies, namely, LC3B antibody (Novus biologicals, United States, Cat No. NB100-2220), p70S6 (Cell signaling #9202), and b-Actin (Sigma, United States, Cat# A2228) at 4°C. The next day, the membrane was washed three times for 10 min with 1X TTBS and then incubated with a secondary antibody diluted to 1:10,000 (Invitrogen, United States) at room temperature for 1 h. The membrane was developed using Western ECL Substrate (Bio-Rad) and visualized using a CCD camera (ChemiDoc, Bio-Rad), and staining was compared to the molecular weight marker using Image Lab Software 5.2.1 build 11 (Bio-Rad). Blots were normalized against b-Actin blotted on the same membrane for hydrogen peroxide and on GAPDH for glutamate treatment. Western blots were analyzed using Image J as described in Hellsten et al. (2018).

### In-Cell ELISA Assay

Measurement of intracellular p53 levels was monitored using Colorimetric Multispecies In-Cell ELISA Kit (Cat No. 62216) as per the protocol of the manufacturer.

### Data Analysis

Mean and SD was calculated from all replicates of each experiment. All analyses were performed with Graph Pad Prism version 5. *t*-tests and two-way ANOVA analysis were performed with appropriate *post-hoc* tests for multiple comparisons for each experiment, as mentioned in figure legends.

## Results

### Effects of Chemically Induced Oxidative Stress on KO Primary Cortex Cells

**FIGURE 1:**
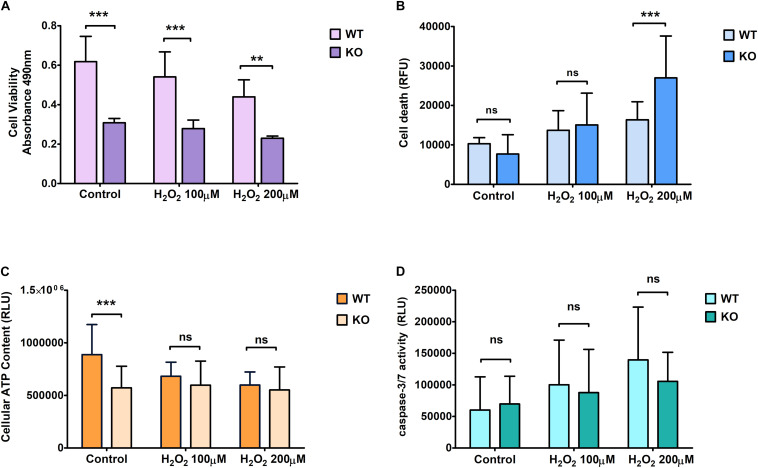
Effect of hydrogen peroxide treatment at different concentrations on primary cortical cells of WT and KO. Cell viability **(A)**, Cell death **(B)**, ATP levels **(C)**, and Caspase 3/7 **(D)** activity was measured using various cell plate-based assays. Graphs represent mean with SD of 18–25 data points from independent cell culture wells. Two-way ANOVA was performed with the Bonferroni *post-hoc* test to compare KO and WT for cells exposed to oxidative stress with significance levels (** ≤ 0.01, and *** ≤ 0.001).

We found that KO cells are significantly less viable than WT cells measured with proliferation assay under controlled conditions and the results also showed that KO cells have significantly less viability than WT cells under oxidative stress (Figure 1A). On the other hand, KO cells are more resistant to stress treatment since the viable cell number is not affected to the same extent as the WT cells under stressed conditions (Figure 1A). When cell death was measured using cytotoxicity LDH assay (Figure 1B), the results showed similar levels of cell death for KO and WT cells in controlled conditions (Figure 1B).

On the other hand, KO cells showed an increase in death under hydrogen peroxide treatment (Figure 1B). ATP levels were measured to assess mitochondrial activity and metabolically active cells under controlled and stressed conditions (Figure 1C). We found that the ATP levels were significantly lower in KO cells than WT cells under controlled conditions. However, ATP levels were not affected by H_2_O_2_ stress in KO cells, while it was reduced in WT cells, reaching the same low levels as the KO cells when exposed to the highest dose of H_2_O_2_.

The relative luminescence was measured to check for Caspase 3/7 activity, which is based on the principle of cell lysis. We found no significant difference in caspase 3/7 activity between WT and KO cells under any conditions. The difference between controlled and stressed conditions showed a similar increment rate for both WT and KO cells (Figure 1D).

### Effects of Chemically Induced Glutamate Excitotoxicity on KO Primary Cortical Cells

**FIGURE 2:**
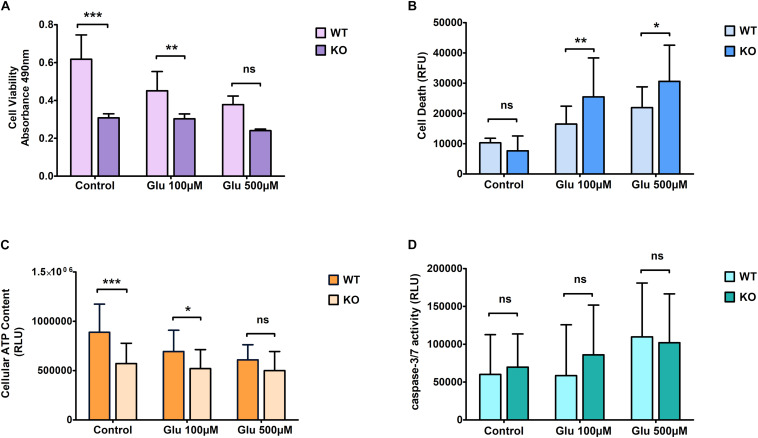
Effect of glutamate excitotoxicity at different concentrations on primary cortical cells of WT and KO. Cell viability **(A)**, Cell death **(B)**, ATP levels **(C)**, and Caspase 3/7 activity **(D)** was measured using various cell plate-based assays. Graphs represent mean with SD of 18–25 data points from independent cell culture wells. Two-way ANOVA was performed with Bonferroni *post-hoc* test to compare KO and WT for cells exposed to glutamate toxicity with significance levels (* ≤ 0.05, ** ≤ 0.01, and *** ≤ 0.001).

We found a trend similar to what we saw for hydrogen peroxide treatment regarding cell viability when cells were exposed to glutamate stress. KO cells showed more resistance under treated conditions compared to WT cells (Figure 2A). Cell death was measured using the LDH assay. KO cells showed an increased death rate under glutamate excitotoxicity (Figure 2B) compared to controlled conditions. The amount of cell death was significantly higher in KO cells than WT cells. ATP levels were reduced slightly more in WT cells than in KO cells when exposed to glutamate stress, reaching a level approximately similar to that of KO cells at the highest dose of exposure (Figure 2C). As for H_2_O_2_ stress, we found no significant difference in caspase 3/7 activity between WT and KO cells (Figure 2D). The bars in the graphs for control cells are the same, which are displayed in Figures 1, 2.

### Unaltered Effect of KO Cells on Mitochondrial Membrane Potential Under Glutamate and Oxidative Stress Treatment

**FIGURE 3:**
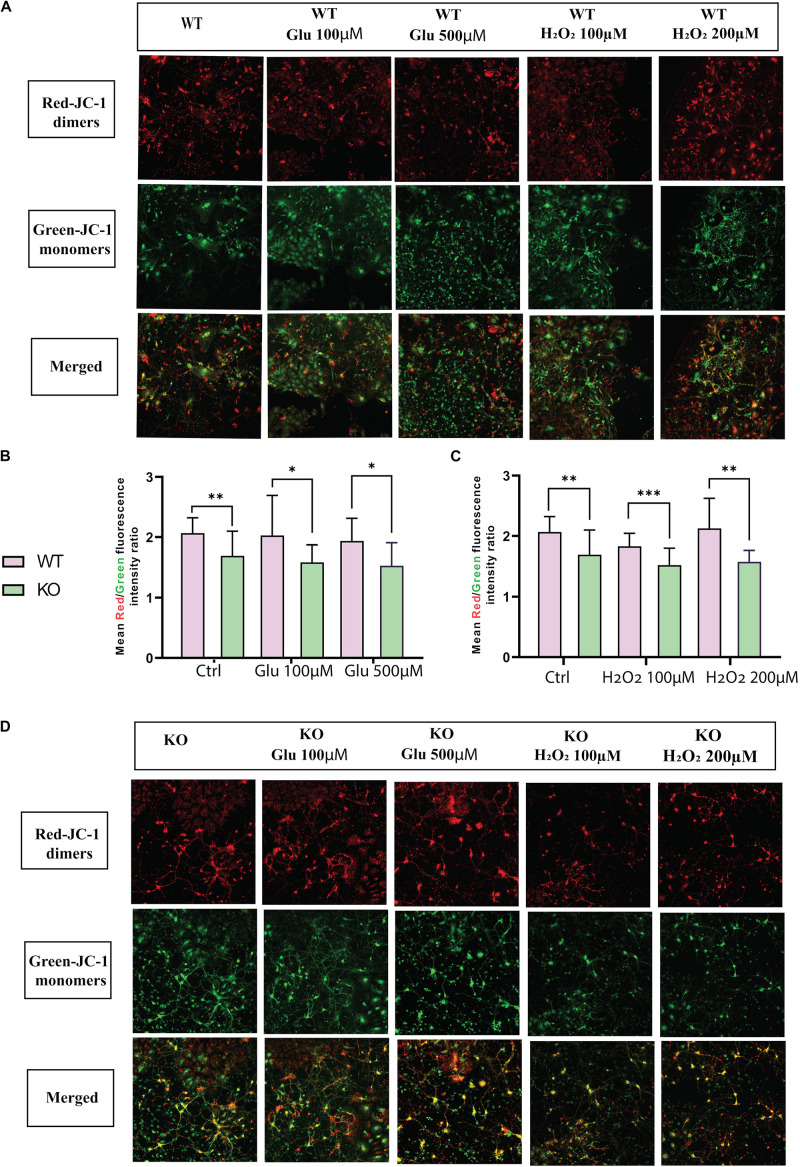
MMP ΔΨm was measured in primary cortex cells of WT **(A)** and KO **(D)** under different treatments of glutamate and hydrogen peroxide using the JC-1 dye. Images were captured using ImageXpress from a molecular device. The fluorescence intensity was measured for both JC-1 green for J-monomers or red for J-aggregates. The graph represents the means of the ratio of fluorescence intensity of red aggregates/green monomer per image for WT and KO primary cortex cells under glutamate toxicity **(B)** and hydrogen peroxide **(C)** treatments. Unpaired *t*-test was performed to compare KO and WT for cells exposed to glutamate excitotoxicity **(B)** and oxidative stress **(C)** with significance levels (^∗^ ≤ 0.05, ^∗∗^ ≤ 0.01, and ^∗∗∗^ ≤ 0.001).

Mitochondrial dysfunction is a crucial feature of unhealthy cells and is a sign of apoptosis. Therefore, mitochondrial membrane potential (MMP) was measured using the fluorescent dye JC1. Monomer JC-1 showed green fluorescence and low MMP due to apoptotic cells, indicating mitochondrial dysfunction. Healthy cells showed higher MMP and formed JC-1 aggregates that are visible as red fluorescence. Thus, mitochondrial membrane depolarization can be measured by a ratio of red to green fluorescence. The fluorescent images showed mitochondrial activity in WT (Figure 3A) and KO (Figure 3D) cells under hydrogen peroxide and glutamate treatments. We found that KO cells showed lower MMP levels than WT cells under both stress treatment (hydrogen peroxide and glutamate) and control conditions (Figures 3B,C). This data shows that there is a possibility of mitochondrial dysfunction in KO cells.

### Protein Expression of LC3B and p70S6 Under Hydrogen Peroxide Treatment

**FIGURE 4:**
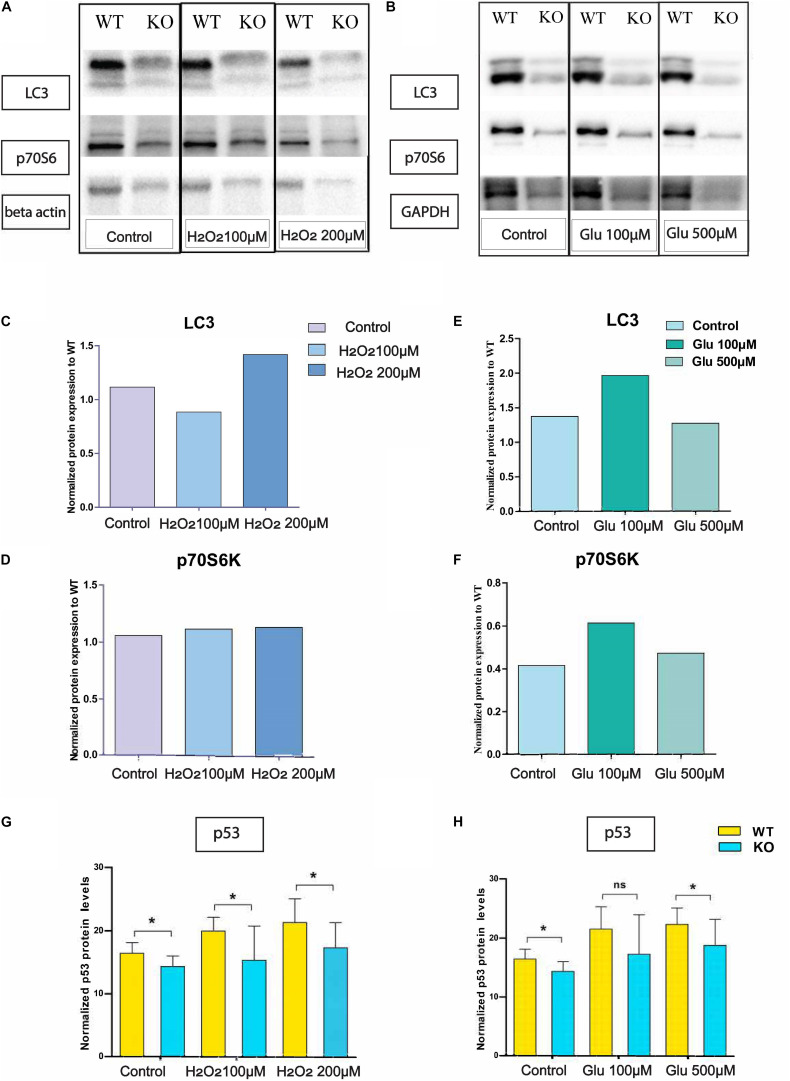
Altered protein levels of protein expression in primary cortex cells of WT and KO under stress. Immunoblot data of antibody LC3 and p-70S6 on hydrogen peroxide **(A)** treated primary cortex cells from WT and KO. The graph represents the ratio of protein expression of LC3 **(C)** and p70S6 **(D)** in KO primary cortex cells compared to WT cells after normalization with respect to loading controls (beta-actin). Immunoblot data of antibody LC3 and p-70S6 under glutamate treated primary cortex cells from WT and KO **(B)**. The graph represents the ratio of protein expression of LC3 **(E)** and p70S6 **(F)** in KO primary cortex cells compared to WT cells after normalization with respect to loading controls (GAPDH). Intracellular p53 protein expression was measured using an In-Cell ELISA p53 kit. The graph represents means with SD, and an unpaired *t*-test was performed to compare KO and WT for cells exposed to oxidative stress **(G)** and glutamate excitotoxicity **(H)** with significance levels (* ≤ 0.05).

We monitored LC3 and p70S6 protein on KO primary cortex cells after hydrogen peroxide treatment (Figure 4A) and glutamate treatment (Figure 4B). There was no change in p70S6 protein in KO (Figure 4D) cells under hydrogen peroxide stress, but under glutamate treatment, p70S6 protein expression increased at 100 μM concentration and returned to the basal level at 500 μM glutamate concentration (Figure 4F). Interestingly, under 100 μM hydrogen peroxide treatment, LC3 expression was 20% lower compared with WT (Figure 4C). However, under glutamate stress at 100 μM of glutamate concentration, LC3 protein expression increased. However, under treatment with a higher concentration of glutamate, LC3 levels reached the same as basal levels of LC3 in KO primary (Figure 4E).

### SLC38A10 KO Alters Intracellular p53 Level in KO and WT Primary Cortex Cells

Intracellular p53 levels were significantly lower in KO primary cortex cells compared with WT in controlled and glutamate exposed conditions. However, at a concentration of 100 μM, the effect is non-significant, although the trend remains the same (Figure 4H). Further, the same trend on p53 expression remains under H_2_O_2_ exposure (Figure 4G).

## Discussion

SLC38A10 is an amino acid transporter, mainly transporting glutamate, glutamine, and aspartic acid (Hellsten et al., 2017). SLC38A10 is localized in the ER and Golgi in neuronal and other cells (Tripathi et al., 2019). It was suggested that SLC38A10 plays a vital role in the regulation of neurotransmission (Hellsten et al., 2017), and polymorphisms in SLC38A10 are associated with autism spectrum disorders (Celestino-Soper et al., 2011), bipolar disorder, schizophrenia, and Alzheimer disorder in human (Guan et al., 2019). Many amino acid transporters are known to participate in redox homeostasis maintenance (Liu et al., 2020). However, there is no information available on the role of SLC38A10 on neuronal cell viability and its possible effect on acute oxidative and glutamate stress. In this study, we try to understand the role of SLC38A10 in cell survival and viability under acute stress.

Oxidative stress and glutamate toxicity are involved in the neuropathology of several neurodegenerative disorders. Glutamate, as a primary neurotransmitter in the brain, is used in around 40% of the synapses. Therefore, increased glutamate levels can result in excessive activation of glutamate receptor signaling, leading to excitotoxicity, and is connected with a wide range of acute and chronic neurodegenerative disorders (Coyle and Puttfarcken, 1993; Sheldon and Robinson, 2007). In earlier studies of glutamate-induced cytotoxicity, various cell lines and primary cortical cells have been utilized (Kritis et al., 2015), and the glutamate transporter GLT1 and GLAST have been shown to link neurological disorders with oxidative and glutamate stress (Sheldon and Robinson; Olivares-Bañuelos et al., 2019). Abnormal glutamate levels and glutamate-glutamine cycle imbalances are one of the main causes of neurodegenerative diseases (Behrens et al., 2002), and it is therefore essential to understand the underlying function of glutamate transporters and their possible role as vital players in neurodegenerative disorders.

SLC38A10 is a known glutamate transporter, which could alter glutamate levels, which is known to affect the viability of neurons. It is known that abundant extracellular glutamate activates continuous depolarization in neurons to induce glutamate toxicity (Lewerenz and Maher, 2015; Mattson, 2019). Several neurodegenerative conditions such as Huntington’s disease, Alzheimer’s disease, lateral amyotrophic sclerosis, Parkinson’s disease, stroke, and traumatic brain injury are associated with excitotoxicity (Hunt et al., 2010; Fendt and Verstreken, 2017).

We found that the cell viability of KO primary cortical cells is lower compared with WT, whereas under acute oxidative and glutamate stress, KO cells remain, in principle, unaffected. We found reduced levels of p53 in KO cells, which can be the underlying cause of their increased stress resistance. A lot of evidence supports that p53 can increase ROS and promote cell elimination and death. p53 supports cell adaptation and survival in response to different stress, such as limited periods of nutrient starvation and hydrogen peroxide, glutamate, and ROS exposure to support survival (Coyle and Puttfarcken, 1993). In neurons, p53 is associated with acute and progressive neurological conditions (Xiang et al., 1996), and loss of p53 in the liver results in changed redox homeostasis and increased DNA damage (Tedeschi and Di Giovanni, 2009).

Previously, p53 has been shown to be involved in amino acid metabolism in cancer cells, specifically with a role in glutamine homeostasis (Tajan et al., 2018). There are several SLCs involved in amino acid homeostasis connected to neuronal disorders (Kandasamy et al., 2018). In cancer cells, the arginine transporter, SLC7A3, is regulated by p53 under glutamine starvation and participate in metabolic regulation of nutrient stress (Lowman et al., 2019). Recent studies have revealed that SLC7A11 promotes cystine uptake and glutathione biosynthesis, resulting in protection from oxidative stress and ferroptotic cell death, and plays critical roles in regulating the glucose and glutamine dependency of cancer cells (Koppula et al., 2018). Similarly, the transporters, SLC35A1 and SLC30A1, act as opposite regulators in cell survival. These transporters play a role in modulating cellular apoptotic response induced to oncolytic virus infections (Moskovskich et al., 2019). We observed similar results after removal of SLC38A10 primary cortex cells, where it does play a role in cell survival under stress. Also, similar to a previous study on primary cortical neurons in culture, homozygote p53 KO mice exhibit little or no response or adverse effects to glutamate stress (Xiang et al., 1996). These results corroborate the findings that KO primary cortex cells remain unaffected in stress. Also, in the present study, we showed that SLC38A10 KO cells have lower p53 expression, which could in part be the cause of the increased resistance to excitotoxicity and H_2_O_2_ challenge.

## Conclusion

SLC38A10 potentially has a role in the adaptation of primary cortex cells under acute stress. The absence of the SLC38A10 gene resulted in changed p53 levels and affected the mitochondrial function. Also, p53 helps to support cell adaptation and survival in response to stressors (Wang et al., 2014). This study establishes a possible correlation of SLC38A10 in cell survival, linked with p53, in primary cortex cells. Thus, there is a need to investigate in detail the role of SLC38A10 amino acid transporter in cellular health.

## Data Availability Statement

The original contributions presented in the study are included in the article/Supplementary Material, further inquiries can be directed to the corresponding author/s.

## Ethics Statement

The animal study was reviewed and approved by (3 5.8.18-09820/2018).

## Author Contributions

RT wrote and drafted manuscript, planned and performed experiments, carried out analysis of data, prepared figures, performed interpretation of data and results, and wrote parts of the manuscript. TA planned and performed cell plate assay, western blot experiments, analysis part of data, and wrote parts of the manuscript. RF obtained funding for the project, supervized the project, performed data analysis and data interpreting, and wrote parts of the manuscript. All authors have read and approved the manuscript and helped with the interpretation of the results.

## Conflict of Interest

The authors declare that the research was conducted in the absence of any commercial or financial relationships that could be construed as a potential conflict of interest.

## Footnotes

1 https://www.mousephenotype.org
