# Supplementary material for: SLC38A10 Transporter Plays a Role in Cell Survival Under Oxidative Stress and Glutamate Toxicity
Source: Front Mol Biosci. 2021 May 5;8:671865. doi: 10.3389/fmolb.2021.671865 (PMC8133219; doi:10.3389/fmolb.2021.671865)
Supplement: Supplementary file 1 [file Table_1.DOCX]

**Supplementary Figure 1**


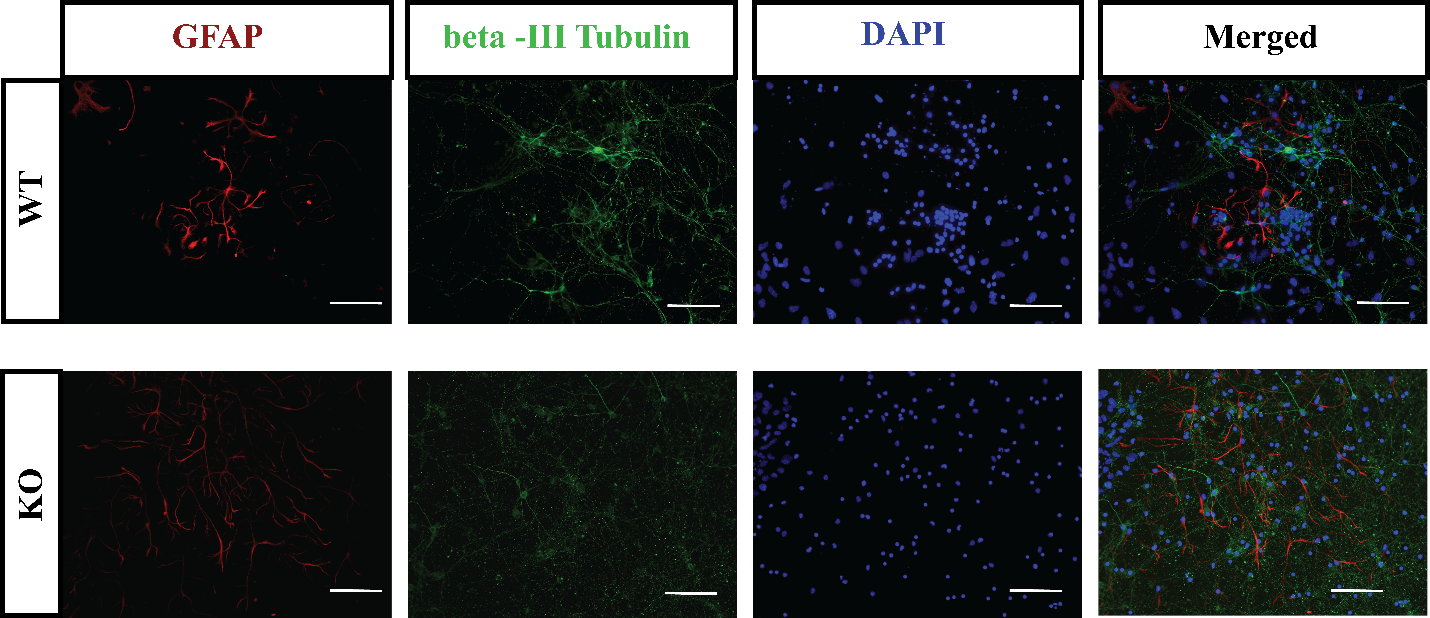


Primary cortex cells derived from WT and KO mice and cultured for seven days. Immunocytochemistry was performed as described in (1) with Anti-Tubulin Antibody, beta III isoform(MAB1637, Sigma-Aldrich) diluted 1:500 in supermix blocking solution and Anti-GFAP antibody (ab134436,abcam) diluted 1:2000. Images were acquired Images were taken at 20x using on an Olympus microscope BX53 with an Olympus DP73 camera and Cell Sens Dimension software further merged and process using Image J (Fiji edition, <https://imagej.net/Fiji/>).

1. E Lekholm; E Perland; MM Eriksson; S V. Hellsten; FA Lindberg; J Rostami; R Fredriksson. Putative membrane-bound transporters MFSD14A and MFSD14B are neuronal and affected by nutrient availability. *Front Mol Neurosci* 10 (2017)
